# Genome-Wide Analysis of the PvHsp20 Family in Switchgrass: Motif, Genomic Organization, and Identification of Stress or Developmental-Related Hsp20s

**DOI:** 10.3389/fpls.2017.01024

**Published:** 2017-06-09

**Authors:** Haidong Yan, Ailing Zhang, Jing Chen, Xiaoyan He, Bin Xu, Guanqi Xie, Zhiming Miao, Xinquan Zhang, Linkai Huang

**Affiliations:** ^1^Department of Grassland Science, Animal Science and Technology College, Sichuan Agricultural UniversityChengdu, China; ^2^College of Agro-Grassland Science, Nanjing Agricultural UniversityNanjing, China

**Keywords:** switchgrass, genome-wide analysis, *Hsp20*, small HSPs, abiotic stress, expression profile

## Abstract

Hsp20 proteins exist in all plant species and represent the most abundant small heat shock proteins (sHSPs) in plants. Hsp20s were known as chaperones maintaining cellular homeostasis during heat or other kinds of abiotic stresses. The objective of this study was to understand the phylogenetic relationship, genomic organization, diversification of motif modules, genome localization, expression profiles, and interaction networks of switchgrass (*Panicum virgatum* L.) Hsp20s (PvHsp20s). A total of 63 PvHsp20s were identified with their consensus as well as unique ACD motifs and gene structures analyzed. Most *PvHsp20*s (87%) were responsive to heat and other kinds of abiotic stresses. When under optimum growth condition, 38 of them displayed relative higher expression levels in inflorescence and seeds, suggesting their protective roles in the stress-sensitive reproductive organs. An *in silico* analysis of interaction network of PvHsp20 proteins further revealed potential interactive proteins, including stress-inducible ones in the network. Furthermore, *PvHsp20* genes unevenly distributed in two sets of homeologous chromosomes, and only segmental duplication was found among the paralogous gene pairs, reflecting that the allotetraploidization of switchgrass allowed the accumulation of *PvHsp20*s that in turn facilitated its successful adaptation in hot and dry plateaus of North America. The present results provided an insight into *PvHsp20*s with an emphasis on the uniqueness of this gene family in switchgrass. Such information shall also be useful in functional studies of *PvHsp20* genes and molecular breeding of switchgrass.

## Introduction

Plants are often exposed to a variety of abiotic stresses such as heat, cold, drought, and salt, etc. Particularly, heat stress causes billion dollar losses of agricultural crops worldwide and is expected become more severe in the future due to the increment of global warming (Deryng et al., 2014). Plant growth, development, and productivity are all adversely affected by heat stress and irreversible damage to plant physiological functions is often observed under heat stress as well (Zhang et al., 2006; Atkinson and Urwin, 2012).

The sessile plants employ a set of molecular elements to adapt to or survive over heat stress, among which heat shock proteins (HSPs) were the most well studied ones that, as one of large stress-related protein families, play a significant role in plants as molecular chaperones maintaining cellular homeostasis in cells under adverse and/or optimal growth conditions (Wang et al., 2004; Timperio et al., 2008; Zhu, 2016). Most HSPs function by aiding protein folding and refolding under stress conditions, protein assembly, translocation, and degradation in cellular processes to maintain stabilization of proteins and membranes (Mayer and Bukau, 2005; Zhang, 2013).

The HSPs could be classified into five families based on their molecular weights: Hsp100s, Hsp90s, Hsp70s, Hsp60s, and Hsp20s (Sarkar N. K., 2009). Hsp20s, or small heat shock proteins (sHSPs), have molecular sizes ranging from 15 to 42 kDa, functioning as molecular chaperones to keep the stability of proteins in an ATP-independent manner, that is crucial for cellular thermotolerance (Guo et al., 2015). Another cardinal characteristic of Hsp20 proteins is the presence of a highly conserved 80 to 100 amino acid sequence, referred to as the alpha crystallin domain (ACD), seated in the proteins C-terminal region, and this ACD has two consensus sequences at its C- and N-terminals with 29 aa and 27 aa in length, respectively (Sarkar N. K., 2009). The ACD domain is characterized by a compact β-strand structure and constitutes two conserved regions including CRI with β2, β3, β4, and β5, and CRII with β6, β7, β8, and β9 (Van et al., 2001; Stamler et al., 2005). It was found that β-strands have different functions. For examples, β2-strand was related to structure dimerization in cases of HSP16.5 in *Methanocaldococcus jannaschii* and HSP16.9 in wheat; the β7-strand plays a crucial role in protein dimer formation in human (Haslbeck and Vierling, 2015; Pandey et al., 2015); and β6 is significant for oligomerization and dimer formation by strand swapping (Van et al., 2001). The arginine in β7-strand and P-G doublet between β3 and β4, were confirmed to be related to human pathologies (Siddique et al., 2008). The ACD domain containing proteins include Hsp20s and the so named Acd proteins. And it is worth to note that biological functions of Hsp20s are different from Acd proteins (Scharf et al., 2001; Sarkar N. K., 2009).

Although Hsp20s are universally present in lower and higher organisms, the number and complexity of Hsp20s are particularly high in the sessile higher plant species than in movable animals that there are only two Hsp20s in the budding yeast and 10 in human (Elicker and Hutson, 2007) when comparing to 19 Hsp20s in *Arabidopsis thaliana* (Scharf et al., 2001), 13 in barley (*Hordeum vulgare* L.) (Pandey et al., 2015), 23 in rice (Sarkar N. K., 2009), 27 in wheat (*Triticum aestivum* L.) (Pandey et al., 2015), 35 in pepper (*Capsicum annuum* L.) (Guo et al., 2015), 42 in tomato (*Lycopersicon esculentum* Mill.) (Yu et al., 2016), and 51 in soybean [*Glycine max* (Linn.) Merr.] (Lopescaitar et al., 2013). Among the 51 Hsp20s in soybean, 47 of them were found responsive to heat shock stress (Lopescaitar et al., 2013). Hsp20 family proteins co-evolved with higher plants along with their diversification and adaptation to different stress environments.

Switchgrass (*Panicum virgatum* L.), as a perennial warm-season C4 model grass, is a highly versatile grass native to North America. Its high biomass yield with minimum demand of inputs is highly desirable for biomass production (Hoogwijk et al., 2003). However, according to predications on climate change, there would be large variations in switchgrass productivity overtime due to increased temperature and accompanied drought stress (Behrman et al., 2013). Current experimental studies also showed that switchgrass biomass yields were greatly reduced under controlled or mimic high temperatures (Hartman and Nippert, 2013; Kandel et al., 2013). Therefore, it is important to understand and reveal molecular elements (e.g., Hsp20s) that contributing to the heat tolerance of switchgrass. However, comprehensive analysis of Hsp20 proteins is not reported in switchgrass yet.

In this study, we conducted a genome-wide comprehensive analysis on switchgrass Hsp20s. Publicly available genomic and transcriptomic databases of switchgrass were employed to systematically analyze Hsp20 protein family and to identify candidate ones contributing to stress tolerance. Such comprehensive knowledge of Hsp20s will ultimately help molecular design or breeding of switchgrass to improve its biomass yield under harsh environmental conditions.

## Materials and methods

### Identification of Hsp20 proteins of switchgrass

Genome and protein sequences were downloaded from the phytozome database (http://phytozome.jgi.doe.gov) and the HMMER software (http://hmmer.janelia.org) was used to build the switchgrass protein data. In addition, the Hidden Markov Model (HMM) file of PvHsp20 (PF00011) domains were downloaded from Pfam (Pfam; http://pfam.sanger.ac.uk/) (Finn et al., 2014) for the identification of Hsp20 proteins from local database (*E* < 0.001). All hits were confirmed by Pfam (PF00011) and NCBI Conserved Domain Search (http://www.ncbi.nlm.nih.gov/Structure/cdd/wrpsb.cgi). The confirmed PvHsp20s were aligned using Clustal X (v 2.0) (Larkin et al., 2007) to remove the redundant sequences. The longest translated protein was picked out among PvHsp20s with alternative splicing sites, and the duplicated result was removed in phylogenetic tree analysis. The Hsp20 protein sequences in rice were referenced according to Sarkar's study (2009), while Hsp20 protein sequences of *Arabidopsis* were obtained from Scharf's (2001) study.

### Gene structure, motif, and phylogenetic tree analysis

The coding sequence (CDS), exons and introns number, amino acid (aa), and chromosomal location information of switchgrass PvHsp20 proteins were retrieved from the phytozome database (http://phytozome.jgi.doe.gov). The Hsp20 proteins' molecular isoelectric point (pI) and weight (Da) were calculated by using the ExPASy program (http://web.expasy.org/compute_pi). Exon-intron display was conducted through the gene structure display server (http://gsds.cbi.pku.edu.cn). The conserved motifs among subgroups of PvHsp20 proteins were identified using the program MEME (Multiple Expectation Maximization for Motif Elicitation; version 4.11.1; http://meme-suite.org/tools/meme) with default parameters, and the maximum number of motifs to find was set to 10 for the prediction of subdomains (Bailey, 2006). The ACD sequences of ACD-containing proteins (Hsp20 and Acd proteins) were aligned via Promals3D structural alignment program (http://prodata.swmed.edu/promals3d/promals3d.php) (Pei et al., 2008). The neighbor-joining (N-J) phylogenetic tree of PvHsp20 proteins of switchgrass, *Arabidopsis*, and rice was built with alignments using ClustalX (bootstrap 1,000 replicates) via MEGA 5.0 (version 5.0, http://www.megasoftware.net) (Tamura et al., 2011).

### Construction of chromosome location images

The chromosomes in switchgrass were ordered to match syntenic fortail millet (*Setaria italica* L.) chromosome order (http://phytozome.jgi.doe.gov). We used MapInspect software (http://mapinspect.apponic.com/) (Ma et al., 2013) to generate chromosome location images to localize switchgrass *PvHsp20* genes. The ratio between nonsynonymous and synonymous nucleotide substitutions (Ka/Ks) were obtained through DNAsp5 software (http://www.ub.edu/dnasp/) (Librado and Rozas, 2009).

### Gene expression analysis for transcripts levels in switchgrass tissues and developmental stages

For each of the ACD-containing proteins (Hsp20 and Acd proteins) identified in switchgrass, Unitranscript IDs were found in the PviUTs database (http://switchgrassgenomics.noble.org/) (Zhang et al., 2013). The integrated transcript sequence database was recognized through searching Unitranscript IDs in PviGEAs (http://switchgrassgenomics.noble.org/) (Zhang et al., 2013). The results from the database were graphically presented in a heatmap format using a log_2_fold change after value normalization through the R Project software (http://miyoviqo.tha.im/).

### Switchgrass affymetrix microarray data analysis under heat stress

Data from the ArrayExpress repository under the accession number E-MTAB-1897 (Li et al., 2013) were retrieved, for the heat-responsive transcription analysis of the *PvHsp20* genes. A total of 92 ACD-containing proteins (Hsp20 and Acd proteins) retrieved from the array data were presented in a heatmap with log_2_ fold change after value normalization by the R Project software (http://miyoviqo.tha.im/) (Ripley, 2001).

### Prediction of PvHsp20s protein-protein interaction network

An interaction network of PvHsp20 proteins was built to analyze genome-wide protein-protein regulation network on the basis of orthologous rice proteins to predict the relationship between PvHsp20s and other proteins by using the rice interactions viewer (http://netbio.sjtu.edu.cn/riceppinet/search.html) (Liu et al., 2017). And then the homologs of these interactive PvHsp20 proteins in switchgrass were identified by using BLAST analysis. The interaction network of PvHsp20 proteins was drawn by Cytoscape_v3.4.0 (Smoot et al., 2011).

### Plant material, growth condition, and stress treatments

Switchgrass cv. Alamo seeds were sown in pots (0.2 meter diameter × 0.3 m tall) containing 1,000 g soil (pH 5.56, 1.35% organic qualitative content, 100.33 mg/kg N, 4.93 mg/kg P, and 332.25 mg/kg K). Growing in a growth chamber (Wenjiang, Sichuan, China) at 28°/20°C (day/night), the plants had photoperiod of 16 h/8 h (day/night). After germination, seedlings of switchgrass were thinned to four plants *per* pot. Fifty days after sowing, the potted switchgrass seedlings were exposed to a variety of stresses including ABA, drought, cold, and salt conditions as follows. For ABA treatment, the seedlings were sprayed with 100 mmol ABA for 16 days and leaves were sampled after 0, 8, and 16 days of treatment. For drought treatment, the potted seedlings were kept without watering for 28 days and at the end of drought treatment, the soil water content of drought-stressed plants was measured to be 10%. And the leaf samples were collected after 0, 14, and 28 days under drought treatment. For cold treatment, the seedlings were subjected to cold stress for 6°C for 28 days and leaves were harvested after 0, 14, and 28 days of treatment. For salinity treatment, the seedlings were watered with 250 mmol/l NaCl for 28 days, and leaves were sampled after 0, 14, and 28 days of treatment. For heat treatment, the plants were exposed to high temperature for 38°C for 28 days and leaves were collected at 0, 14, and 28 days. All materials that harvested from each treatment were frozen in liquid nitrogen immediately and stored at −80°C before for RNA isolation. With three biological replicates, all experiments were conducted three times for qRT-PCR analysis.

### RNA isolation, cDNA synthesis, and real-time qRT-PCR

Total RNA kit II (Qiagen, USA) was used to isolate total RNA. RNA concentration measurement, DNaseI treatment, and cDNA synthesis were implemented as described in our previous study (Huang et al., 2014). Nine primer pairs of predicted stress-related genes were designed via Primer 5 software (Lalitha, 2004; Table S1). To confirm the primer specificity, we blasted each primer sequence to the switchgrass genome (https://phytozome.jgi.doe.gov/pz/portal.html). We also confirmed whether the nine primer pairs displayed corresponding melting curves with a single sharp peak and an electrophoresis pattern of a single amplicon with precise length. Besides, the *UCE2* gene, as a reference gene, helped to calculate the expressions of nine genes (Huang et al., 2014). Moreover, the cut-off value was set to 2-fold for stress-specific expression as “down-regulated” or “up-regulated” ones.

## Results

### Identification and phylogenetic analysis of PvHsp20 proteins

**Figure 1:**
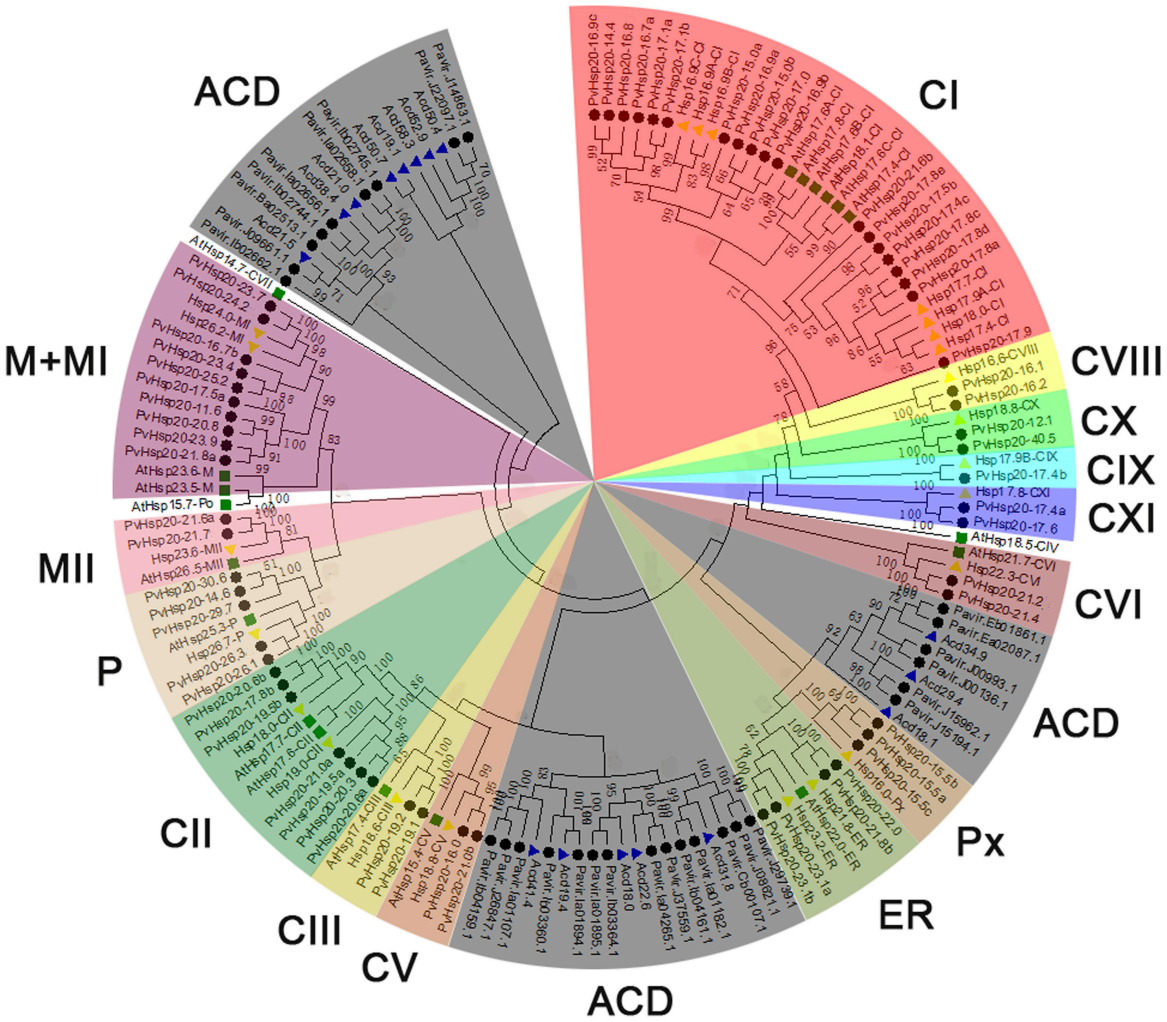
Phylogenetic pie chart of Hsp20 proteins in switchgrass, rice, and *Arabidopsis*. The Hsp20 proteins are used for building the phylogenetic tree via MEGA 5.0 after the alignments by ClustalX. The unrooted neighbor-joining analysis was conducted with 1,000 bootstrap replicates and p-distance method (only percentage bootstrap scores above 50% were shown in this tree). Rice, *Arabidopsis*, and switchgrass are Hsp20 proteins were marked with yellow triangles, green squares, and black circles, while Acds in rice were marked with blue triangles. The 15 distinct subfamilies are marked by different colors, and the Acds subgroup was colored in gray.

**Figure 2:**
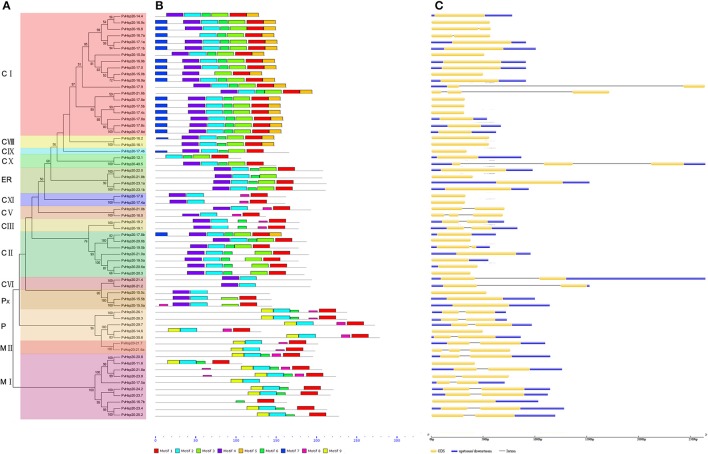
The phylogenetic relationships, motif compositions, and gene structure for sHsp proteins in switchgrass. **(A)** The alignments of 63 PvHsp20 proteins were conducted by ClustalX, and the phylogenetic tree was built by using MEGA 5.0 based on Neighbor-joining method with 1000 bootstrap replicates and p-distance method. The percentage bootstrap scores above 50% were shown in this tree. 14 phylogenetic sub-clusters were divided according to Figure 1 with different color backgrounds. **(B)** Schematic representation of the conserved motifs in sHsp proteins was displayed by MEME. Each motif is represented by a type of colored box, while the black lines indicate the non-conserved sequences. **(C)** Exon/intron organizations of sHsps. Exons and introns are yellow boxes and black lines, respectively, and upstream/downstream sequences are blue boxes.

A total of 92 proteins containing ACD were identified in switchgrass from the newly released switchgrass genome database (*Panicum virgatum* JGI v1.1), and detailed information about these proteins were presented in Supplementary File 1. A neighbor-joining (N-J) phylogenetic tree constructed with switchgrass, *Arabidopsis*, and rice proteins containing ACD domains clearly showed that 63 out of 92 switchgrass proteins were clustered with Hsp20s (or sHsps), while the rest 29 proteins with Acd proteins (Figure 1). And the 63 Hsp20s proteins were designated according to their molecular weights (Supplementary File 1). The lengths of all proteins ranged from 100 to 360aa with three of them longer than 300aa (Pavir.Ib04161.1, Pavir.J00993.1, and Pavir.J00136.1). The predicted molecular weights of the 92 ACD-containing proteins were between 11.22 kDa (Pavir.J14863.1 and Pavir.J22097.1) and 40.48 kDa (PvHsp20-40.5) with an average of 20.67 kDa. The isoelectric points (pI) of 92 ACD-containing proteins ranged from 5.04 (PvHsp20-14.6) to 11.48 (PvHsp20-15.5c), and 54 of them had pIs lower than 7.0, and 36 had pIs higher than 7.5 (noting that plant cytosol's pH is ~7.5) (Supplementary File 1). In addition, the 63 switchgrass Hsp20s were further classified into 14 subgroups in accordance with their predicated subcellular localizations: 39 Hsp20s in subgroups C (for cytosol/nucleus) I-XI, 3 in Px (peroxisome), 4 in ER (endoplasmic reticulum), 5 in P (plastid), and 12 in M (mitochondria) I-II (Figure 2).

### Gene structures and motifs of PvHsp20s

In a simplified N-J phylogenetic tree containing only PvHsp20s, similar gene structures and motif arrangements were found among those classified in the same clade (subgroup), which consistency in turn supported the reliability of the phylogenetic classification (Figure 2). It was notable that PvHsp20s presumably located in cytosol/nucleus (CI-CX), endoplasmic reticulum (ER), and peroxisome (Px) shared strikingly different motif arrangements from those in plastid (P) and mitochondria (MI-II). And more than half of PvHsp20s (37/63) were intronless in subgroups CVIII, CIX, CXI, ER, and Px, while most proteins in subgroups CIII, CV, CVI, P, and MI-II had one or two introns.

A total of 10 types of consensus motifs were revealed among the switchgrass ACD-containing proteins, where nine types of motifs were found among 63 PvHsp20s (motif logos shown in Figure S1). Motifs-1, -2, -3, -4, and -6 were the most conserved ones (Figure 2). Matching these consensus motifs with featured sequences of ACD, we found that the Consensus Region I (CRI) of ACD was composed of motifs 2, 4, and 6, while CRII was composed of motifs 1 and 3. The presence of all of these five motifs composing the ACD were only found in one half (31 out of 63) of PvHsp20s in subgroups of CI, CII, CVIII, CIX, CX, and ER, while the rest PvHsp20s lacked at least one of these five motifs, suggesting the diversification with the ACD across PvHsp20s, which was proposed as one key domain for protein-protein interaction.

**Figure 3:**
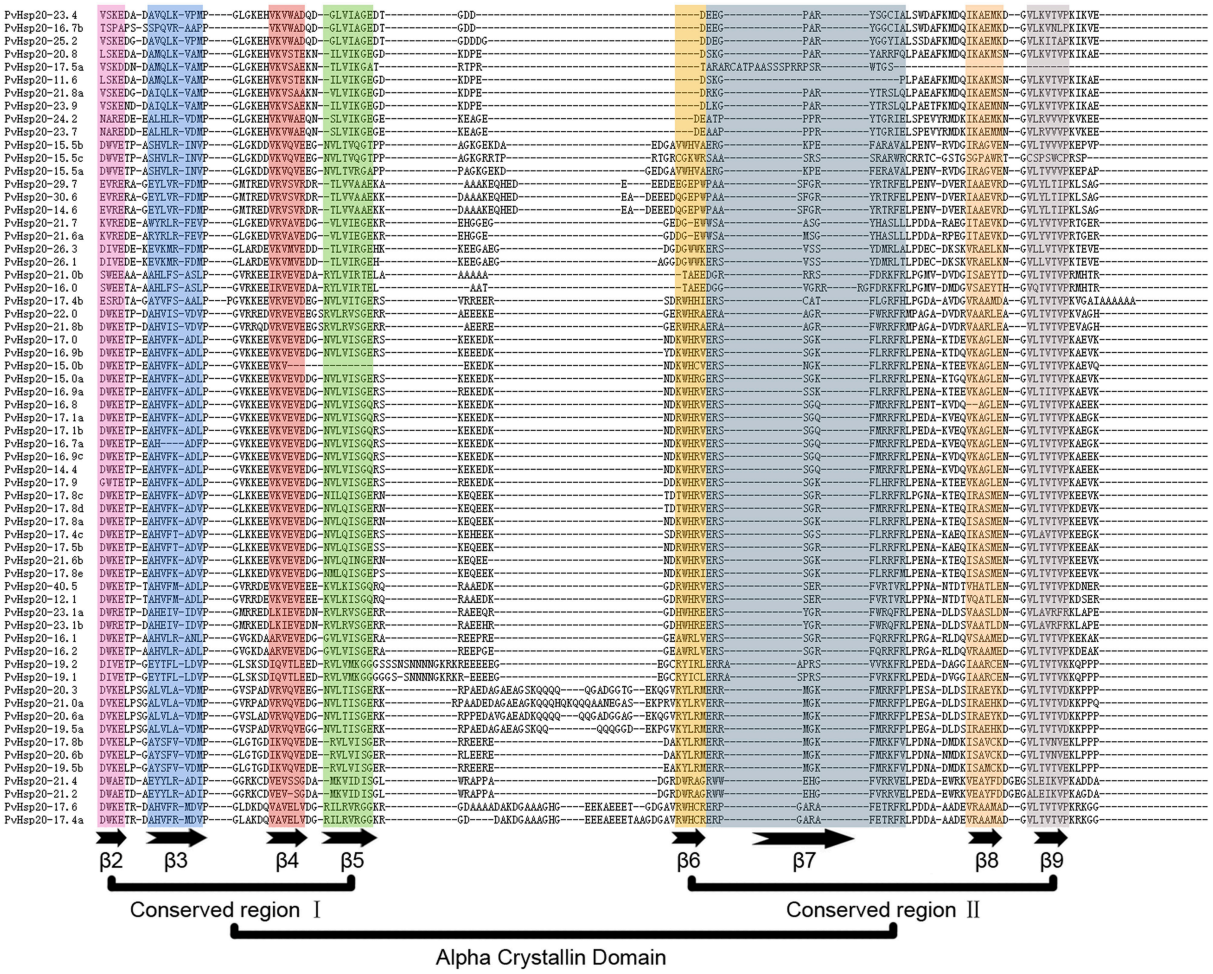
The alignment of ACDs of sHsps in switchgrass. The names of all members were shown in the left side of figure. Each predicted β-plated sheet is shown for different color backgrounds. β2, β3, β4, and β5 were included in conserved region I (CRI), while β6, β7, β8, and β9 were included in conserved region II (CRII).

Multiple alignments of the conserved ACD domains among PvHsp20s were conducted as shown in Figure 3 to illustrate the relationship between consensus motifs and predicated secondary structures of ACDs. The CRI of ACD were composed of motifs 2, 4, and 6 featured with four β-sheets (β2, β3, β4, β5), and CRII were composed of motifs 1 and 3 featured with three β-sheets (β6, β7, β8, and β9). Among the 63 PvHsp20s, only PvHsp20-11.6 lacked β7, PvHsp20-15.0b lost β5, and HSP17.5a missed β8 and β9 (Figure 3). Additionally, a total of 10 PvHsp20s (15.9%) lacked β6. Because β6-strand is significant for dimer formation and oligomerization by strand swapping (Van et al., 2001), the process of dimer formation or oligomerization for these PvHsp20s without β6-strand might be influenced. These PvHsp20s lacking of β6 might depend on other courses to remedy this omission for protein-protein interaction (Waters, 2013).

### Expression patterns of *PvHsp20*s in heat stress

**Figure 4:**
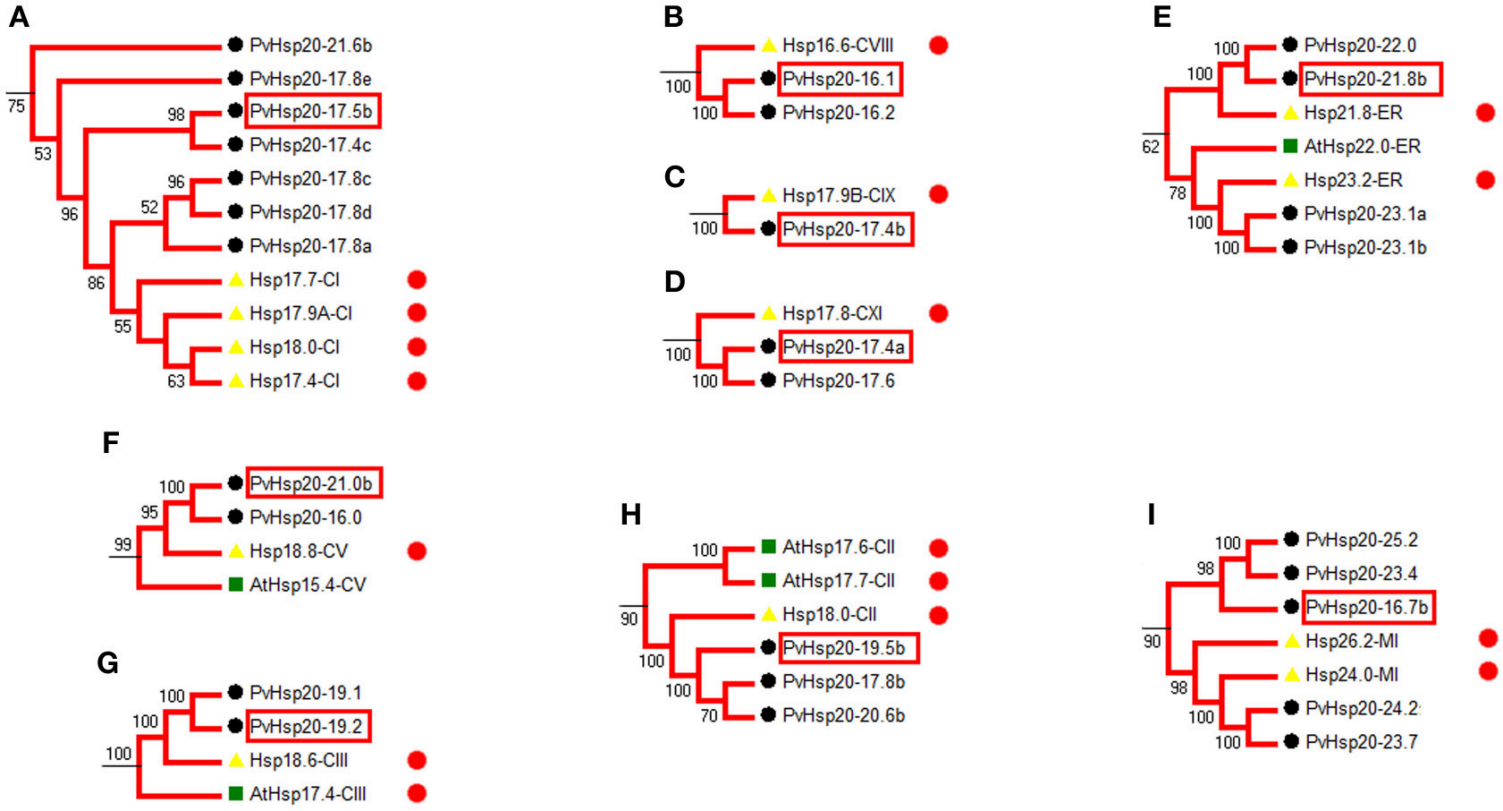
Phylogenetic relationships show the PvHsp20s that are orthologous to annotated stress-responsive Hsp20 genes in rice and *Arabidopsis*. 28 PvHsp20 proteins were predicted to be involved in stress tolerance, and they were divided into nine functional groups (**A–I**). The red circles indicate proteins with stress-related functional annotation (Table S2), and proteins marked with red frames were used to be validated in further qRT-PCR. Rice, *Arabidopsis*, and switchgrass are Hsp20 proteins marked with yellow triangles, green squares, and black circles, respectively.

Heat shock protein (HSP) protein families play a significant role in heat stress tolerance. With reference to functional annotated Hsp20s (Table S2), we identified 28 corresponding orthologous PvHsp20s that were potentially involved in the regulation of abiotic stress tolerance in nine functional groups (Figures 4A–I).

**Figure 5:**
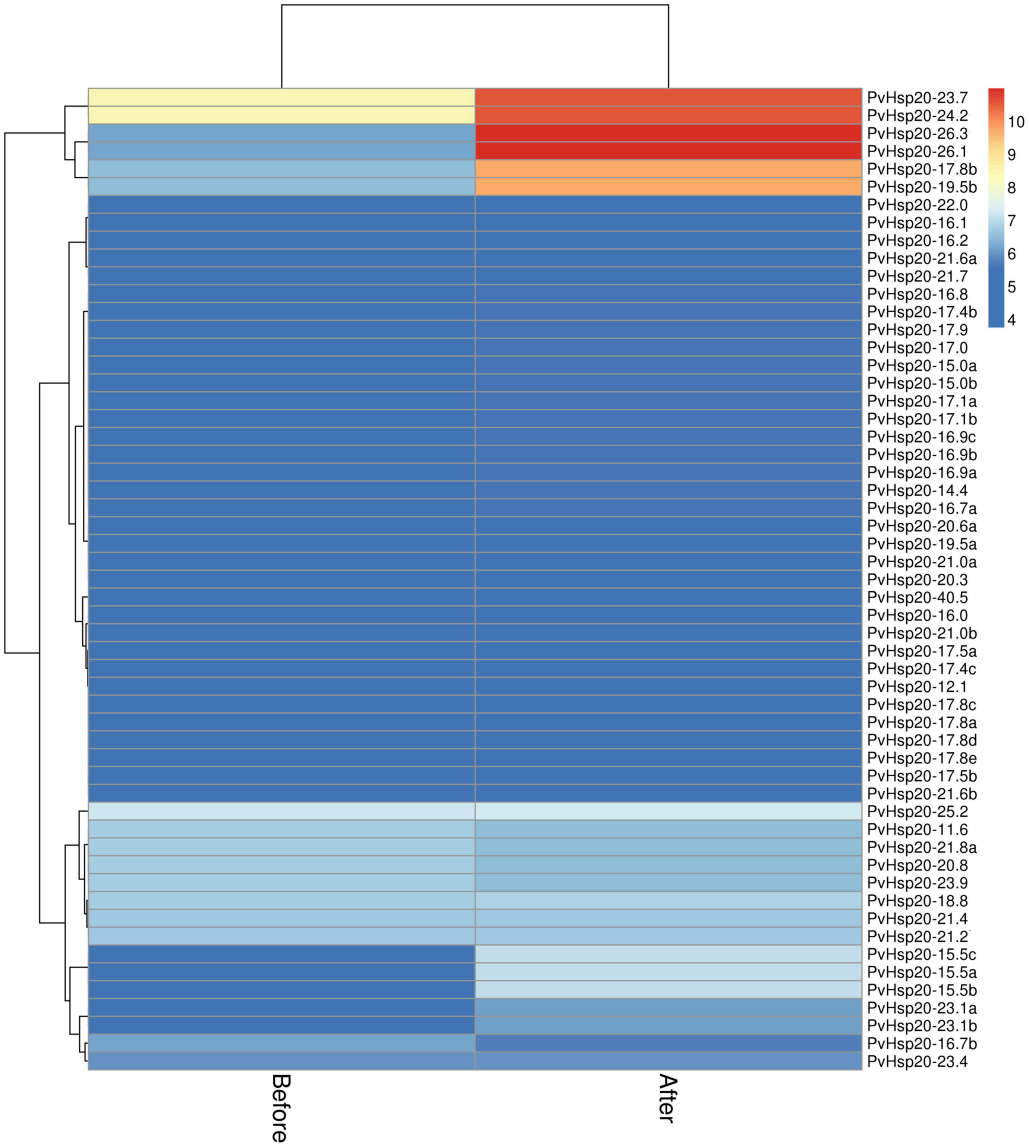
The expression patterns of 55 *sHsp*s upon heat stress based on Affymetrix data (Li et al., 2013).

Furthermore, the expression profiles of *PvHsp20* and *Acd* genes from a switchgrass Affymetrix array (Li et al., 2013) were analyzed to discover those transcriptionally responsive to heat stress. A total of 55 *PvHsp*s (Figure 5) and 22 *Acd*s (Figure S2) were discovered in the switchgrass GeneChip (Supplementary Files 2, 3). Among the 55 *PvHsp*s, 48 were up-regulated, and 24 of them were with more than 1.5-fold change and four with more than 5-fold change after heat stress. As for the *Acd* genes, nearly half of them were down-regulated (9/22, 40.9%), only three were up-regulated, and the rest 11 genes did not display an obvious change (below 1.2-fold change after heat stress) after heat stress. These results were consistent with previous report that most *PvHsp20*s were transcriptionally up-regulated and some *Acd* genes were down-regulated by heat stress (Sarkar N. K., 2009).

Among the 24 up-regulated *PvHsp20*s with more than 1.5-fold change after heat stress, seven of them (*PvHsp20-17.4b, -23.1a, -23.1b, -19.5b, -17.8b, -24.2*, and *-23.7*) were orthologous to previously functional annotated Hsp20s in model plant species (Table S2), further suggesting that these *PvHsp20s* might be involved in plant heat tolerance.

### Expression patterns of *PvHsp20*s in different tissues and developmental stages

**Figure 6:**
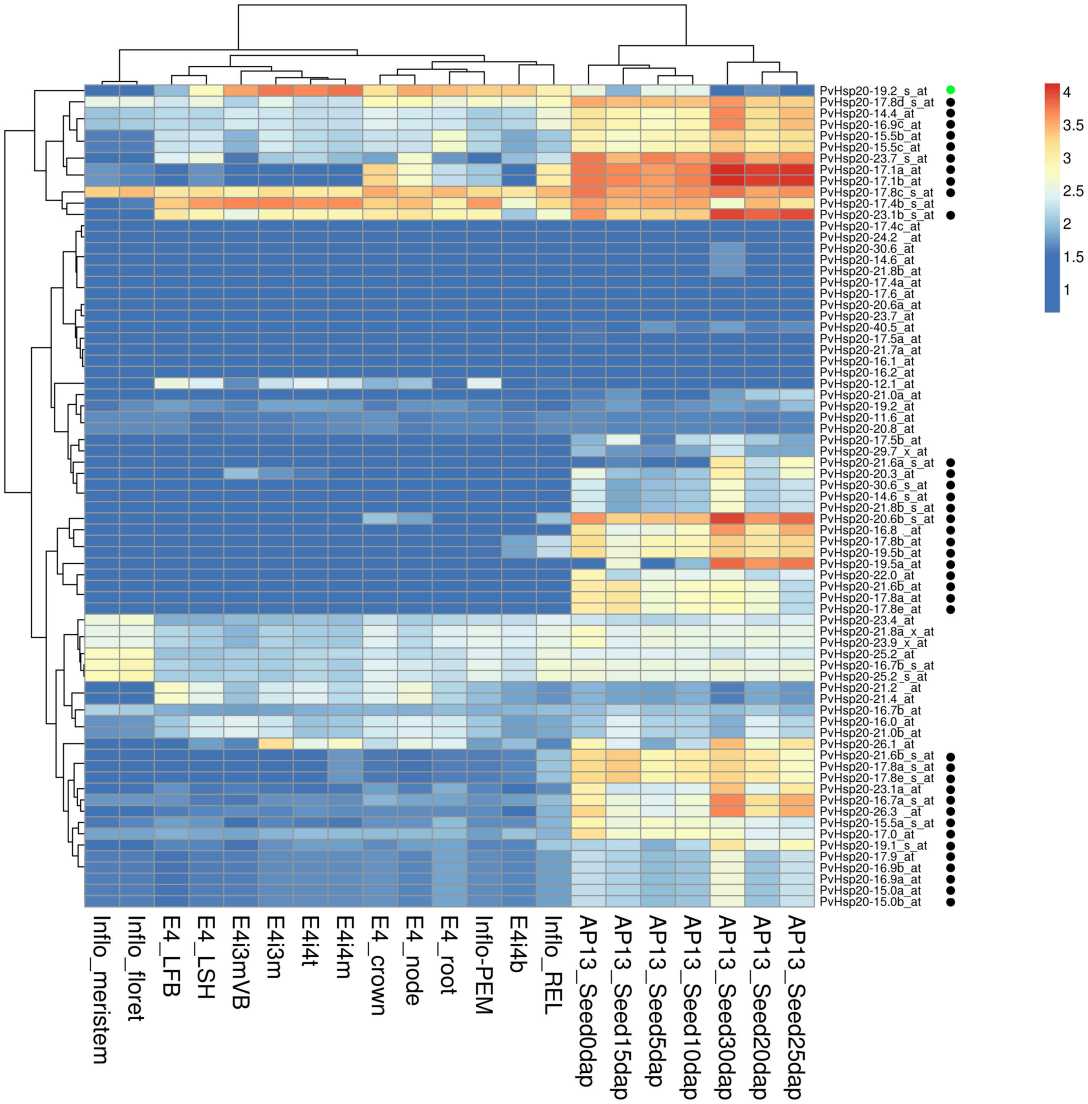
Heatmap of expression patterns of *sHsps* in 21 organs, tissues or at different developmental stages. E4-root, E4-crown, E4-node, E4-LFB, and E4-LSH indicate whole root system, whole crown, pooled nodes, pooled leaf blade from plant, and polled leaf sheath, respectively. E4i3m and E4i3mVB mean Middle 1/5 fragment of the 3rd internode and vascular bundle isolated from 1/5 fragment of the 3rd internode. E4i4b, E4i4t, and E4i4m indicate bottom 1/5 fragment of the 4th internode, top 1/5 fragment of the 4th internode, and middle 1/5 fragment of the 4th internode 4. Inflo-meristem, Inflo-floret, Inflo-REL, and Inflo-PEM indicate inflorescence meristem (0.5–3.0 mm), floret of inflorescence when glumes are 10–20 mm, rachis and branch elongation of inflorescence (50–150 mm), and panicle emergence of inflorescence (>200 mm), respectively. AP13_Seed0d, AP13_Seed5d, AP13_Seed10d, AP13_Seed15d, AP13_Seed20d, AP13_Seed25d, AP13_Seed30d represent whole flowers at anthesis stage, whole seeds 5 days post fertilization, whole seeds with visible caryopsis, whole seeds at the milk stage, whole seeds at the soft dough stage, whole seeds at the hard dough stage, whole seeds at the physiological maturity stage, respectively. The black and green circles besides each gene name indicate these genes were specifically expressed in seed developments and lignified tissues, respectively.

In the absence of stress, differential expression of *Hsp20*s during development stages and/or in different tissues has also been recorded before (Vierling, 1991). Therefore, *PvHsp20*s' expression patterns in 21 different tissues and developmental stages were analyzed using switchgrass Gene Expression Atlas (PviGEA). According to the analysis, a total of 39 *PvHsp20*s had differential expression patterns in different organ/tissues. (Figure 6). Specifically, 38 genes displayed relatively higher expression levels in reproductive organs (inflorescence and seeds at different developmental stages) (Figure 6). These accumulations of *PvHsp20*s might contribute to the thermotolerance in reproductive organs (florets and seeds), and indicated that *PvHsp20s*' significance in normal plant growth and development even under optimal growth condition. As for the *Acd* genes, none of them was found with tissue-specific expression pattern (Figure S3).

To our knowledge, there was no report concerning Hsp20s' involvement in secondary cell wall strengthening and/or lignification. However, the gene expression altas showed that one *PvHsp20* gene (*PvHsp20-19.2*) was highly expressed in lignified tissues (e.g., crown, roots, node, internode, and inflorescence branches) (Figure 6). Concerning the importance of lignin content in this lignocellulosic biomass grass, it would be interesting to further understand the function of *PvHsp20-19.2*.

### qRT-PCR analysis of selected abiotic stress-responsive *PvHsp20*s

**Figure 7:**
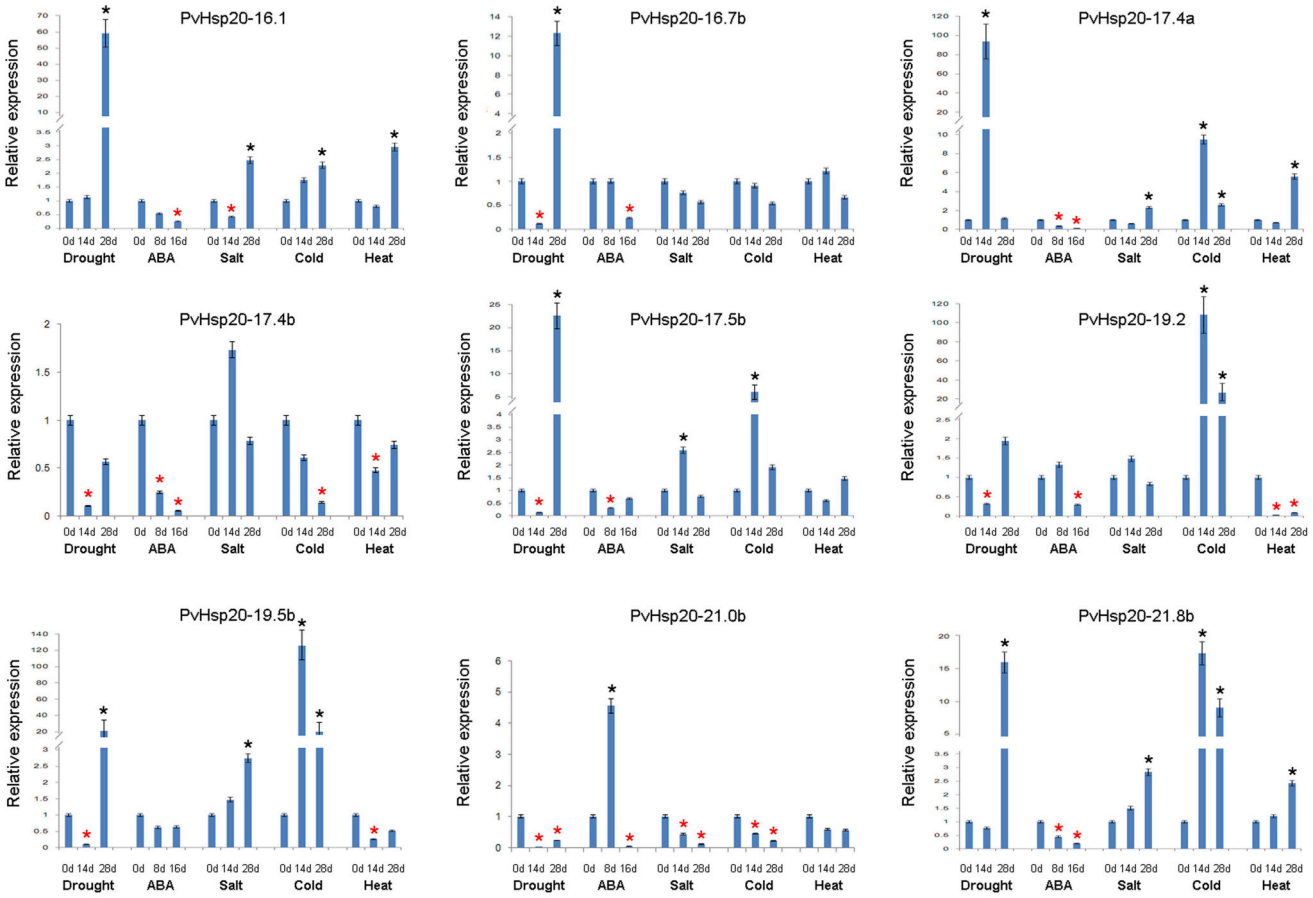
The expression profiles of nine selected *PvHsp20*s in Figure 4 by using qRT-PCR. The relative expression of each *PvHsp20*s were normalized via reference gene *UCE2* in different stresses. The red * represents the expression of treatment groups is below half of the control groups (0 d), while the black ^*^ indicates the expression is more than twice to control group (0 d).

Based on *in silico* data analyses described above, expression profiles of nine *PvHsp20*s that were predicted as stress-related genes and/or orthologues of functional-annotated *Hsp20* genes, were further studied under drought, ABA, salt, cold, and heat treatments using qRT-PCR (Figure 7; Supplementary File 4). Setting the cut-off value at 2-fold change, eight out of nine selected *PvHsp20* genes were expressed at significantly higher or lower levels when treated with at least one of these abiotic stresses. Among these *PvHsp20*s, only three genes (*PvHsp20-16.1, -17.4*, and *-21.8b*) were transcriptionally up-regulated by heat stress, and another three (*PvHsp20-17.4b, -19.2*, and *-19.5b*) down-regulated by heat stress, respectively. On the other hand, all of the tested *PvHsp20*s were responsive to drought treatment, among which five were significantly up-regulated by under severely drought stress (drought treatment for 28 days), one was up-regulated under moderate drought stress (drought treatment for 14 days), and three were significantly down-regulated under moderate drought condition. When exposed to salt treatment, five genes containing *PvHsp20-16.1, -17.4a, -17.5b, -19.5b*, and *-21.8b* were significantly up-regulated after 14 and 28 d, while only one gene (*PvHsp20-21.0b*) were significantly depressed. Cold treatment significantly induced expression of six out of nine tested genes except *PvHsp20-16.7b, -17.4b*, and *-21.0b*. In particular, *PvHsp20-19.2* and *-19.5b* increased over 20 fold change after 14 and 28 d of cold treatment. Yet, the up-regulation of *PvHsp20* genes to these abiotic stresses seemed to be independent on ABA that nearly all of these were down-regulated in response to ABA treatment, except *PvHsp20-21.0b*.

### The interaction network of PvHsp20 proteins

**Figure 8:**
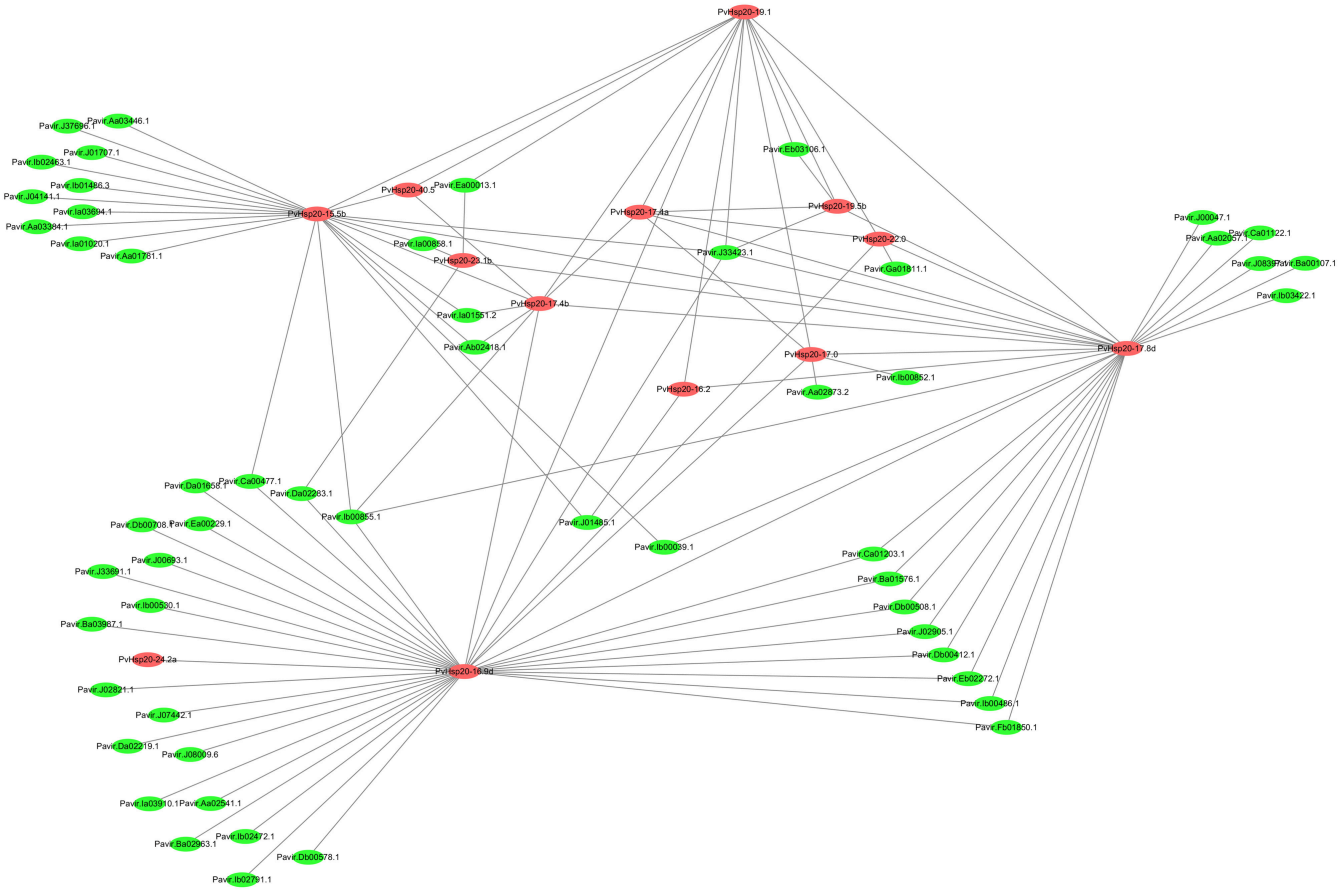
Interaction network of PvHsp20 proteins and other switchgrass genes orthologous to rice. The line thickness were the combined score.

PvHsp20s function as chaperones. Therefore, an interaction network of PvHsp20 proteins was built on the basis of orthologous rice proteins to predict the relationship between PvHsp20s and other proteins in switchgrass (Figure 8; Supplementary File 5). Nearly all abiotic stress-related PvHsp20s as described above (84.6%, 11/13) were built in this network. A total of 109 high confidence interactive relationships (score > 0.85) and 68 interactive proteins were discovered, including 13 PvHsp20s and 55 other switchgrass proteins. The interaction network of PvHsp20 proteins displayed a complex functional relationship. All PvHsp20 proteins directly or indirectly interacted with each other, and according to the results from Table S2 and affymetrix array (Figure 5). PvHsp20-16.9d was predicted to directly interact with the largest number (35) of proteins in switchgrass, followed by PvHsp20-17.8d (27), PvHsp20-15.5b (22), and PvHsp20-19.1 (13), indicating their core status of stress regulation in the protein interaction. In addition, PvHsp20-15.5b, -19.1, -17.8d, and -16.9d all interacted with one drought inducible protein (Pavir.J33423.1) encoding a DnaJ superfamily chaperone (Ye et al., 2016).

### Chromosomal localization of *PvHsp20*s

**Figure 9:**
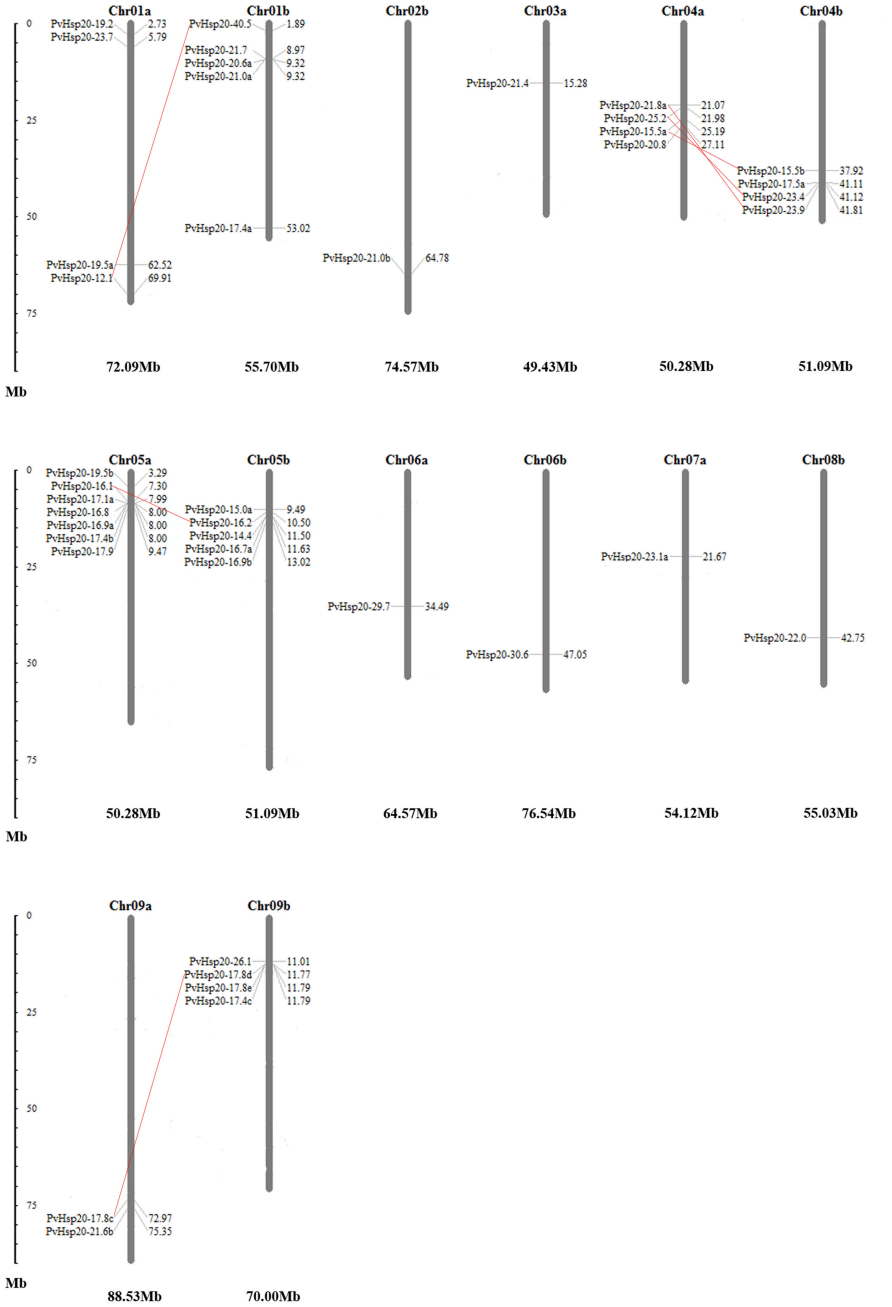
Chromosomal locations of 41 *PvHsp20*s. Duplications generated by allotetraploidy were connected by full red lines. The numbers besides each gene name were locations of these genes, and the larger number indicated the *PvHsp20s* were closer to end of the chromosome. The number below each chromosome was the whole length for this chromosome.

Allotetraploid switchgrass has two subgenomes designated as A and B (Okada, 2010). In this study, chromosomal localizations of 41 *PvHsp20*s were further analyzed. As shown in Figure 9, these genes were unevenly distributed on 14 chromosomes of five homeologous pairs (Chr01a/b, Chr04a/b, Chr05a/b, Chr06a/b, and Chr09a/b) and two nonhomologous chromosomes (Chr02b, Chr03a, Chr07a, and Chr08b). For example, there was only one gene (14.63%) located on chromosomes 02b, 03a, 06a/06b, 07a, and 08b; while there were seven genes (17.07%) on chromosome 05a. A total of 6 pairs of paralogous ACD-containing genes (bootstrap value >95 in the phylogenetic tree) with defined chromosomal locations were linked with red line in Figure 9, and all of them were in homeologous chromosomes (Table 1). Tandem gene duplication, defined as paralogous genes physically linked in tandem with less than five genes in-between, was not found in this study, suggesting that these paralogous ACD-containing genes were all duplicated due to the allotetraploidy event of switchgrass. During the time course of evolution, favorable mutations leading to species divergences were usually fixed (called “diversifying selection”), while those causing disadvantages were eliminated (called “purifying selection”). Comparison of synonymous and nonsynonymous substitution rates between the six pairs of *PvHSP20* paralogous genes suggested that 83.33% (5 out of 6) gene pairs were under purifying selection, while only one pair (1/6, 16.67%) was under diversifying selection (Table 1).

**Table 1:** Purifying and diversifying selection of *PvHsp20s*.

**Gene name**	**Chromosomal locations**	**Ks**	**Ka**	**Ka/Ks**	**Evolutionary selection**	**Duplication type**
PvHsp20-12.1/40.5	Chr01a/01b	0.0265	0.0083	0.313208	Purifying	Homeologs
PvHsp20-16.2/16.1	Chr05b/05a	0.0887	0.0356	0.401353	Purifying	Homeologs
PvHsp20-15.5b/15.5a	Chr04b/04a	0.0681	0.0324	0.475771	Purifying	Homeologs
PvHsp20-23.9/21.8a	Chr04b/04a	0.0632	0.0494	0.781646	Purifying	Homeologs
PvHsp20-23.4/25.2	Chr04b/04a	0.0402	0.042	1.044776	Diversifying	Homeologs
PvHsp20-17.8c/17.8d	Chr09a/09b	0.1096	0.0166	0.151460	Purifying	Homeologs

## Discussion

Homologous gene analysis with the whole gene family members is a reliable method to predict their potential functions according to known results from related or model species (Rabbani et al., 2003; Le, 2011; Yuan et al., 2015). For instance, *AtHSP25.3-p, AtHSP22.0-ER*, and *AtHSP18.1-CI* in *Arabidopsis*, were involved in plant heat tolerance (Wang et al., 2016). Some of their counterpart rice *Hsp20*s were predicted or experimentally proved to be involved in plant heat stress tolerance as well (Guan et al., 2004; Chang et al., 2007; Sarkar N. K., 2009; Schmidt et al., 2012). The comprehensive analysis of switchgrass *PvHsp20* genes not only provided a meaningful overview of these family genes, but also pinpointed some *PvHsp20* genes that might associate with heat and the other abiotic stresses, including 28 *PvHsp20*s orthologous to functional-annotated *Hsp20*s in model species.

According to the phylogenetic analysis, a total of 63 *PvHsp20*s were analyzed to discover their gene organization which directly reflects the evolution of gene family members (Xu et al., 2012). Specifically, 37 *PvHsp20*s are intronless (pattern 1), 22 contain one intron (pattern 2), and only four genes include two introns (pattern 3). Most *PvHsp20*s in the CI and ER subgroups had no intron, which was consistent with those in pepper, rice and soybean, but the gene structure (exon-intron) of CII group in switchgrass was different from those in these species (Ouyang et al., 2009; Lopescaitar et al., 2013; Guo et al., 2015), indicating that the intron pattern might not be well conserved across different species. When we associate the expression pattern of *PvHsp20*s (Affymetrix array data) with their respective intron numbers, we found that among the 24 up-regulated *PvHsp20*s with more than 1.5 fold expression changes after heat stress, 16 were intronless, and eight had only one intron (Table S3). The observation was in agreement with previous reports that the absence of intron or short intron length was shown to increase the gene expression in plants (Chung et al., 2006; Ren et al., 2006). During evolution of eukaryotes, extensive intron loss or gain happened due to stochastic accumulation of introns in huge eukaryotic genomes originated from intron-poor ancestors during their evolution (Jeffares et al., 2006). And genes that lost their introns tend to be rapidly activated under stress (Jeffares et al., 2008). Therefore, the gene organization (exon-intron structure) of *PvHsp20*s might contribute to their transcriptional regulation under stress condition (Sarkar A., 2009).

Another interesting finding is that 39 *PvHsp20*s displayed tissue-specific expression profiles. Such expression profiles were also recorded with rice, pepper, and *Arabidopsis Hsp20*s (Scharf et al., 2001; Sarkar N. K., 2009; Guo et al., 2015). For examples, eight *Arabidopsis Hsp20* genes were specifically expressed in leave, and some rice *Hsp20*s were specifically accumulated in seeds. In the case of *PvHsp20*s, a majority of them had relatively low expression levels in vegetative organs/tissues under optimum growth condition, but 38 of these genes showed relatively higher expression levels in reproductive organs (inflorescence and seeds at different developmental stages) (Figure 6). Interestingly, among 38 of these *PvHsp20*s with higher expression levels in reproductive organs, 21 of them were heat-inducible according to the Affymetrix array data (Table S3). In addition, the 24 heat stress related sHsps displayed a great number of sHsps in cytoplasmic groups (Table S3), similar to those in rice and *Arabidopsis* (Guan et al., 2004; Swindell et al., 2007). Multiplicity of these genes in cytoplasm might indicate the functional redundancy of cytoplasmic Hsp20s in switchgrass. Considering that plants at early flowering to seed setting stages were more susceptible to high temperature (Saini et al., 1983; Mitchell and Petolino, 1988; Shonnard and Gepts, 1994; Peet et al., 1998), these relative high expression levels of *PvHsp20*s in reproductive organs even without stress indicated that these *PvHsp20*s played vital roles in maintaining the cellular homeostasis during meiosis, fertilization and seed setting.

A total of 63 *PvHsp20*s were identified in this study, which number is about the highest among the reported plant species. It is known that gene duplication is crucial for the generation of novel and advantageous alleles (Vision et al., 2000; Hurles, 2004). Segmental duplication and tandem amplification of chromosomal regions contribute to gene evolution, diversification, as well as genome expansion (Leister, 2004). However, only segmental duplication of *PvHsp20*s was found among the paralogous pairs. And distribution patterns of *PvHsp20*s on the two homeologous chromosomal sets (subgenomes a and b as shown in Figure 8) were quite uneven. It was predicated that the tetraploid switchgrass were derived from two close progenitors due to a recent allotetraploidization event at ~1 million years ago (Mya) (Huang et al., 2003; Yuan et al., 2015). This narrow time frame after the polyploidization event might not be sufficient for gene diversification. On the other hand, the accumulation of *Hsp20* genes could have greatly facilitated the successful colonization of switchgrass in relatively dry and hot southern plateaus in the North America. Therefore, a great number of *PvHsp20* genes persisted in the switchgrass genome and the majority of them were still under purifying selection to retain their functions.

## Conclusion

In this study, we have conducted a genome-wide analysis for all the *PvHsp20*s in switchgrass to reveal their phylogenetic relationship, genomic organization, ACD modules diversification, genome localization, expression profiles, and interaction networks. The present results provided not only an insight into *PvHsp20*s with an emphasis on the uniqueness of this gene family in switchgrass, but also useful information in selecting useful *PvHsp20* genes for further experimental studies for the genetic improvement of switchgrass.

## Author contributions

HY, AZ, JC, XH, GX, and ZM drafted the work and revised the manuscript. BX, LH, and XZ substantial contributed to the conception and design of the work.

### Conflict of interest statement

The authors declare that the research was conducted in the absence of any commercial or financial relationships that could be construed as a potential conflict of interest.
