# Supplementary material for: Genome-Wide Analysis of the PvHsp20 Family in Switchgrass: Motif, Genomic Organization, and Identification of Stress or Developmental-Related Hsp20s
Source: Front Plant Sci. 2017 Jun 9;8:1024. doi: 10.3389/fpls.2017.01024 (PMC5465300; doi:10.3389/fpls.2017.01024)
Supplement: Supplementary file 6 [file Image1.PDF]

	The logo of motif **1-9**	E-value	Sites	Width
**Motif 1**	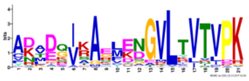	2.8e-733	82	21
**Motif 2**	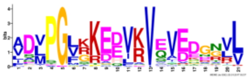	3.5e-699	85	21
**Motif 3**	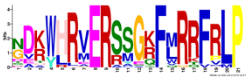	6.8e-432	34	21
**Motif 4**	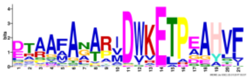	3.5e-351	44	21
**Motif 5**	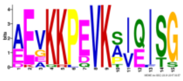	4.5e-143	21	15
**Motif 6**	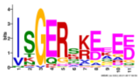	1.8e-139	55	11
**Motif 7**	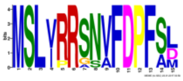	3.7e-127	15	15
**Motif 8**	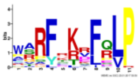	1.3e-077	35	11
**Motif 9**	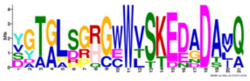	7.3e-047	9	21

Figure S1 Motif information of PvHsp20 proteins.
