# Supplementary figures and images for: Genome-Wide Analysis of the PvHsp20 Family in Switchgrass: Motif, Genomic Organization, and Identification of Stress or Developmental-Related Hsp20s
Source: Front Plant Sci. 2017 Jun 9;8:1024. doi: 10.3389/fpls.2017.01024 (PMC5465300; doi:10.3389/fpls.2017.01024)

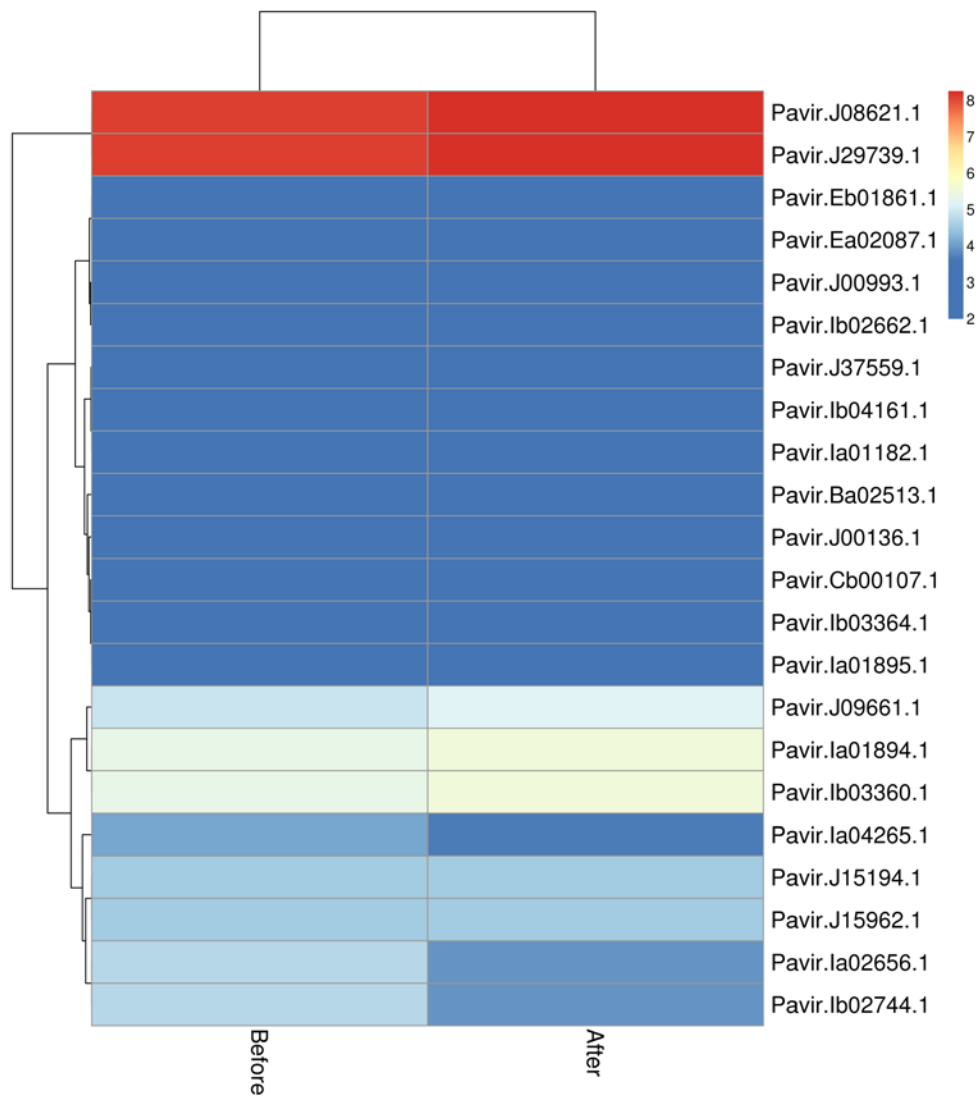

Figure S2 The expression patterns of *Acds* upon heat stress based on Affymetrix data.

Supplement: Supplementary file 7 [file Image2.PDF]
