# Supplementary material for: Genome-Wide Analysis of the PvHsp20 Family in Switchgrass: Motif, Genomic Organization, and Identification of Stress or Developmental-Related Hsp20s
Source: Front Plant Sci. 2017 Jun 9;8:1024. doi: 10.3389/fpls.2017.01024 (PMC5465300; doi:10.3389/fpls.2017.01024)
Supplement: Supplementary file 8 [file Image3.PDF]

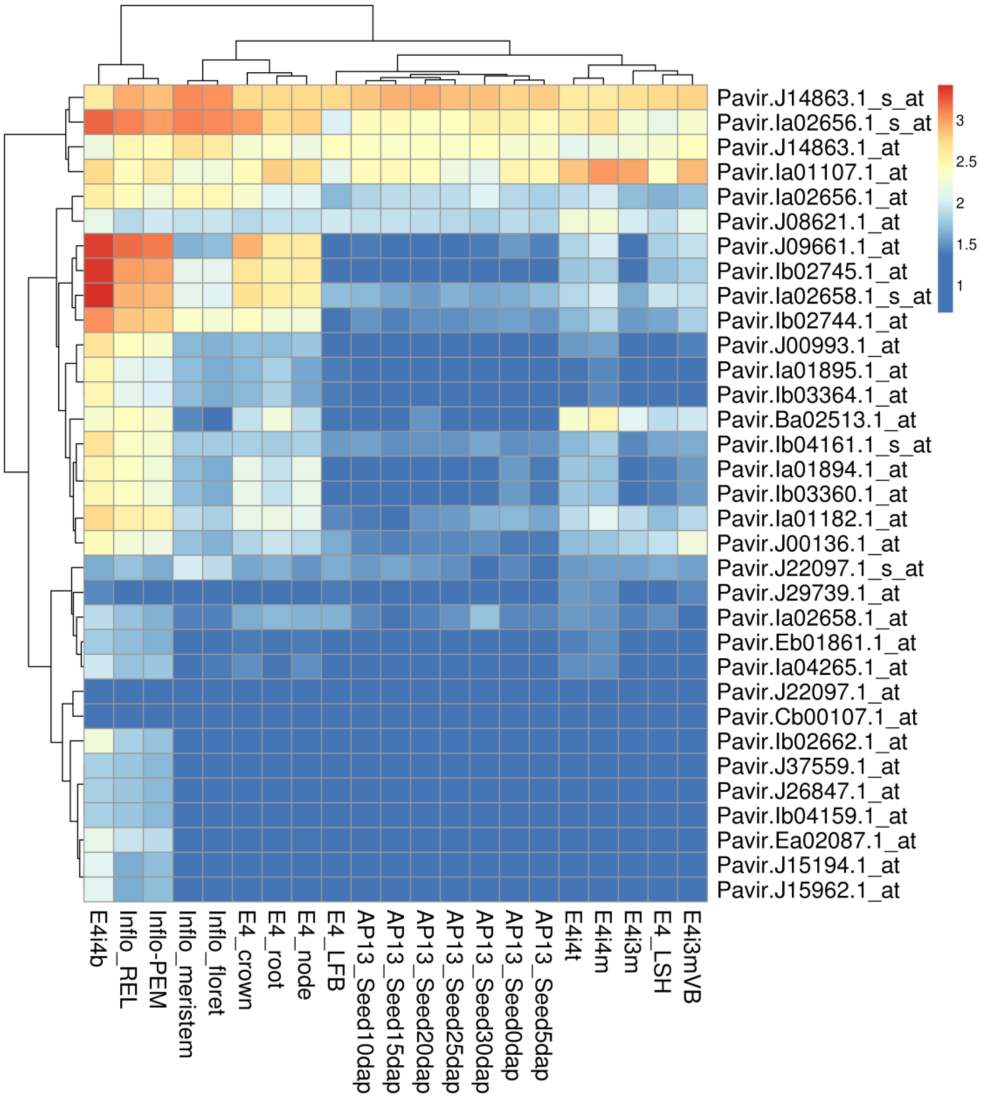

Figure S3 Heatmap of expression patterns of *Acds* in 21 organs, tissues or at different developmental stages. E4-root, E4-crown, E4-node, E4-LFB, and E4-LSH indicate whole root system, whole crown, pooled nodes, pooled leaf blade from plant, and polled leaf sheath, respectively. E4i3m and E4i3mVB mean Middle 1/5 fragment of the 3rd internode and vascular bundle isolated from 1/5 fragment of the 3rd internode. E4i4b, E4i4t, and E4i4m indicate bottom 1/5 fragment of the 4th internode, top 1/5 fragment of the 4th internode, and middle 1/5 fragment of the 4th internode 4. Inflo-meristem, Inflo-floret, Inflo-REL, and Inflo-PEM indicate inflorescence meristem (0.5-3.0 mm), floret of inflorescence when glumes are 10–20 mm, rachis and branch elongation of inflorescence (50–150 mm), and panicle emergence of inflorescence (>200 mm), respectively. AP13_ Seed0d, AP13_ Seed5d, AP13_ Seed10d, AP13_ Seed15d, AP13_ Seed20d, AP13_ Seed25d, AP13_ Seed30d represent whole flowers at anthesis stage, whole seeds 5 days post fertilization, whole seeds with visible caryopsis, whole seeds at the milk stage, whole seeds at the soft dough stage, whole seeds at the hard dough stage, whole seeds at the physiological maturity stage, respectively.
