# Supplementary material for: Genome-Wide Analysis of the PvHsp20 Family in Switchgrass: Motif, Genomic Organization, and Identification of Stress or Developmental-Related Hsp20s
Source: Front Plant Sci. 2017 Jun 9;8:1024. doi: 10.3389/fpls.2017.01024 (PMC5465300; doi:10.3389/fpls.2017.01024)
Supplement: Supplementary file 9 [file Table1.DOCX]

**Table S1** The nine primers of genes used to be validated.

| **Gene** | **Forward primer** | **Reverse primer** |
| --- | --- | --- |
| Hsp20-16.1 | GTGCGGAGCGGCGGCGGCGTGTTCG | CACGTGCGCGGCGGGGGTCTCCTTC |
| Hsp20-16.7b | GTTCTTCTCCGCAGGTACGCGCCGC | GCACCCCGTCCTTCATCTCCGCCTT |
| Hsp20-17.4a | GAGGCGGAGGAGGAGACCGCCGCCG | TGTTGCCGCCGTGGTGCCGCTTCTT |
| Hsp20-17.4b | CGCTGGTAAGGCTGTTGGACA | ACCCTCACCTCCTCCTTCTTCA |
| Hsp20-17.5b | GAGAGCAGCAAGGAGCAGGAAGAGA | ACGGGCTTGACATCGAGCTTCTTAG |
| Hsp20-19.2 | CAGCAGCAGCAACAGCAACAACAAC | CGATGGTGACCTGCACGGACT |
| Hsp20-19.5b | AACGCCGACATGGACAAGAT | ACCTCAGCACTCGCCACAAC |
| Hsp20-21.0b | GCCTTTTCACCCTCCTGTCCTCTCC | CGTCCTCCACCTCCACCCTGATCTC |
| Hsp20-21.8b | AGGCGGACGAGGAGGAGGAT | GCACCTGGATGTTGACGACCTT |
