# Supplementary material for: Genome-Wide Analysis of the PvHsp20 Family in Switchgrass: Motif, Genomic Organization, and Identification of Stress or Developmental-Related Hsp20s
Source: Front Plant Sci. 2017 Jun 9;8:1024. doi: 10.3389/fpls.2017.01024 (PMC5465300; doi:10.3389/fpls.2017.01024)
Supplement: Supplementary file 10 [file Table2.DOCX]

**Table S2** Plant functional-annotated Hsp20 genes and their closest PvHsp20s homolog (s).

| **Gene ID (TIGR)** | **Protein name** | **Gene functional annotation** | **References** | **Closest PvHsp20 homolog(s)** |
| --- | --- | --- | --- | --- |
| At5g12030 | AtHsp17.7-CII | Involved in drought and salt regulation | ([Sun et al., 2001](#_ENREF_7)) | PvHsp20-19.5b/17.8b/20.6b in cluster h |
| At1g54050 | AtHsp17.4-CIII | Involved in heat regulation; seed maturation | ([Takahashi and Komeda 1989](#_ENREF_8); [Wehmeyer et al., 1996](#_ENREF_10)) | PvHsp20-19.1/19.2 in cluster g |
| At4g10250 | AtHsp22.0-ER | Involved in heat regulation | ([Wang et al., 2016](#_ENREF_9)) | None |
| At3g46230 | AtHsp17.4-CI | Involved in heat regulation; Embryo development; Germination | ([Rhoads et al., 2005](#_ENREF_4); [Wehmeyer et al., 1996](#_ENREF_10)) | None |
| At1g53540 | AtHsp17.6C-CI | Involved in heat regulation; seed maturation | ([Helm and Vierling 1989](#_ENREF_3); [Wehmeyer et al., 1996](#_ENREF_10)) | None |
| At5g59720 | AtHsp18.1-CI | Involved in heat regulation | ([Wang et al., 2016](#_ENREF_9)) | None |
| At5g12020 | AtHsp17.6-CII | Involved in Osmotic stress; Germination | ([Sun et al., 2001](#_ENREF_7)) | PvHsp20-19.5b/17.8b/20.6b in cluster h |
| Os01g08860 | Hsp18.0-CII | Involved in heat regulation | ([Chang et al., 2007](#_ENREF_1)) | PvHsp20-19.5b/17.8b/20.6b in cluster h |
| Os01g04370 | Hsp16.9A-CI | Involved in heat regulation | ([Guan et al., 2004](#_ENREF_2)) | None |
| Os01g04380 | Hsp16.9B-CI | Involved in heat, salt, and cold regulation | ([Guan et al., 2004](#_ENREF_2)) | None |
| Os01g04360 | Hsp16.9C-CI | Involved in heat and cold regulation | ([Guan et al., 2004](#_ENREF_2)) | None |
| Os03g15960 | Hsp17.9A-CI | Involved in heat, salt, and drought regulation | ([Guan et al., 2004](#_ENREF_2)) | PvHsp20-17.8d/17.8c/17.8a/17.4c/17.5b/21.6b/17.8e in cluster a |
| Os03g16040 | Hsp17.7-CI | Involved in heat and drought regulation | ([Guan et al., 2004](#_ENREF_2)) | PvHsp20-17.8d/17.8c/17.8a/17.4c/17.5b/21.6b/17.8e in cluster a |
| Os03g16020 | Hsp17.4-CI | Involved in heat, salt, and drought regulation | ([Sarkar 2009](#_ENREF_5)) | PvHsp20-17.8d/17.8c/17.8a/17.4c/17.5b/21.6b/17.8e in cluster a |
| Os03g16030 | Hsp18.0-CI | Involved in cold regulation | ([Sarkar 2009](#_ENREF_5)) | PvHsp20-17.8d/17.8c/17.8a/17.4c/17.5b/21.6b/17.8e in cluster a |
| Os02g54140 | Hsp18.6-CIII | Involved in salt and drought regulation | ([Sarkar 2009](#_ENREF_5)) | PvHsp20-19.1/19.2 in cluster g |
| Os02g52150 | Hsp24.0-MI | Involved in heat, salt, and drought regulation | ([Sarkar 2009](#_ENREF_5)) | PvHsp20-24.2/23.7/16.7b/23.4/25.2 in cluster in cluster i |
| Os06g11610 | Hsp26.2-MI | Involved in heat and salt regulation | ([Sarkar 2009](#_ENREF_5)) | PvHsp20-24.2/23.7/16.7b/23.4/25.2 in cluster in cluster i |
| Os07g33350 | Hsp18.8-CV | Involved in salt regulation | ([Schmidt et al., 2012](#_ENREF_6)) | PvHsp20-21.1b/16.0 in cluster f |
| Os02g48140 | Hsp17.8-CXI | Involved in heat regulation | ([Guan et al., 2004](#_ENREF_2)) | PvHsp20-17.4a/17.6 in cluster d |
| Os11g13980 | Hsp21.8-ER | Involved in heat regulation | ([Guan et al., 2004](#_ENREF_2)) | PvHsp20-23.1a/23.1b/22.0/21.8b in cluster e |
| Os04g36750 | Hsp23.2-ER | Involved in heat regulation | ([Guan et al., 2004](#_ENREF_2)) | PvHsp20-23.1a/23.1b/22.0/21.8b in cluster e |
| Os01g04350 | Hsp17.9B-CIX | Involved in heat regulation | ([Guan et al., 2004](#_ENREF_2)) | PvHsp20-17.4b in cluster c |
| Os01g04340 | Hsp16.6-CVIII | Involved in heat and drought regulation | ([Guan et al., 2004](#_ENREF_2)) | PvHsp20-16.1/16.2 cluster b |
| Os05g42120 | Hsp22.3-CVI | Involved in biotic and oxidative stress | ([Guan et al., 2004](#_ENREF_2)) | PvHsp20-21.4/21.2 |
| Os02g12610 | Hsp19.0-CII | Involved in oxidative stress | ([Guan et al., 2004](#_ENREF_2)) | PvHsp20-20.3/20.6a/19.5a/21.0a |

**Reference**

Chang P., Jinn T.L., Huang W.K., Chen Y., Chang H.M., andWang C.W. (2007) Induction of a cDNA clone from rice encoding a class II small heat shock protein by heat stress, mechanical injury, and salicylic acid. *Plant Sci* 172, 64-75

Guan J.C., Jinn T.L., Yeh C.H., Feng S.P., Chen Y.M., andLin C.Y. (2004) Characterization of the genomic structures and selective expression profiles of nine class I small heat shock protein genes clustered on two chromosomes in rice (*Oryza sativa* L.). *Plant Mol Biol* 56, 795-809

Helm K.W., andVierling E. (1989) An *Arabidopsis thaliana* cDNA clone encoding a low molecular weight heat shock protein. *Nucl Acid Res* 17, 7995

Rhoads D.M., White S.J., You Z., Mrinalini M., andElthon T.E. (2005) Altered gene expression in plants with constitutive expression of a mitochondrial small heat shock protein suggests the involvement of retrograde regulation in the heat stress response. *Physiol Plant* 123, 435-444

Sarkar N.K. (2009) Rice sHsp genes: genomic organization and expression profiling under stress and development. *BMC Genom* 10, 393

Schmidt R., Schippers J.H., Welker A., Mieulet D., Guiderdoni E., andMueller-Roeber B. (2012) Transcription factor OsHsfC1b regulates salt tolerance and development in *Oryza sativa* ssp. japonica. *Aob Plant* 2012, pls011

Sun W., Bernard C., Van d.C.B., Van M.M., andVerbruggen N. (2001) At-HSP17.6A, encoding a small heat-shock protein in Arabidopsis, can enhance osmotolerance upon overexpression. *Plant J Cell Mol Biol* 27, 407–415

Takahashi T., andKomeda Y. (1989) Characterization of two genes encoding small heat-shock proteins in *Arabidopsis thaliana*. *Mol Genet Genom* 219, 365-372

Wang X.Y., Huang W.L., Yang Z.M., Liu J., andHuang B.R. (2016) Transcriptional regulation of heat shock proteins and ascorbate peroxidase by CtHsfA2b from African bermudagrass conferring heat tolerance in *Arabidopsis*. *Sci Rep* 6, 28021

Wehmeyer N., Hernandez L.D., Finkelstein R.R., andVierling E. (1996) Synthesis of small heat-shock proteins is part of the developmental program of late seed maturation. *Plant Physiol* 112, 747-757
