# Supplementary material for: Genome-Wide Analysis of the PvHsp20 Family in Switchgrass: Motif, Genomic Organization, and Identification of Stress or Developmental-Related Hsp20s
Source: Front Plant Sci. 2017 Jun 9;8:1024. doi: 10.3389/fpls.2017.01024 (PMC5465300; doi:10.3389/fpls.2017.01024)
Supplement: Supplementary file 11 [file Table3.DOCX]

**Table S3** The specific expression tissue, intron number, and subcellular classification of 24 up-regulated sHsps with more than 1.5 fold change after heat stress in affymetrix array data.

| **Gene name** | **Tissue** | **Intron number** | **Subcellular classification** |
| --- | --- | --- | --- |
| PvHsp20-15.5a | seed | 0 | Px |
| PvHsp20-15.5b | seed | 0 | Px |
| PvHsp20-15.5c | seed | 0 | Px |
| PvHsp20-14.4 | seed | 0 | CI |
| PvHsp20-26.3 | seed | 1 | P |
| PvHsp20-16.7a | seed | 1 | CI |
| PvHsp20-17.8b | seed | 0 | CII |
| PvHsp20-26.1 | - | 1 | P |
| PvHsp20-23.7 | seed | 1 | MI |
| PvHsp20-16.9a | seed | 0 | CI |
| PvHsp20-16.9b | seed | 0 | CI |
| PvHsp20-16.9c | seed | 0 | CI |
| PvHsp20-24.2 | - | 1 | MI |
| PvHsp20-19.5b | seed | 1 | CII |
| PvHsp20-17.1b | seed | 0 | CI |
| PvHsp20-17.1a | seed | 0 | CI |
| PvHsp20-15.0b | seed | 0 | CI |
| PvHsp20-15.0a | seed | 0 | CI |
| PvHsp20-17.0 | seed | 0 | CI |
| PvHsp20-17.9 | seed | 1 | CI |
| PvHsp20-17.4b | - | 0 | CIX |
| PvHsp20-23.1a | seed | 0 | ER |
| PvHsp20-23.1b | seed | 0 | ER |
| PvHsp20-16.8 | seed | 1 | CI |
